# Efficacy and Safety of Oral Chinese Herbal Medicine for Migraine: A Systematic Review and Meta-Analyses Using Robust Variance Estimation Model

**DOI:** 10.3389/fneur.2022.889336

**Published:** 2022-07-06

**Authors:** Shaohua Lyu, Claire Shuiqing Zhang, Xinfeng Guo, Anthony Lin Zhang, Jingbo Sun, Genghang Chen, Charlie Changli Xue, Xiaodong Luo

**Affiliations:** ^1^The Second Affiliated Hospital of Guangzhou University of Chinese Medicine, Guangdong Provincial Hospital of Chinese Medicine and Guangdong Provincial Academy of Chinese Medical Sciences, Guangzhou, China; ^2^The China-Australia International Research Centre for Chinese Medicine, School of Health and Biomedical Sciences, RMIT University, Melbourne, VIC, Australia

**Keywords:** migraine, Chinese herbal medicine, systematic review, meta-analysis, robust variance estimation

## Abstract

**Background:**

Migraine is a prevalent headache disorder with significant impacts on patients' quality of life and economic burden. Chinese herbal medicine (CHM) is commonly prescribed for migraine in China. This review aimed to provide a rigorous evaluation of evidence on the efficacy of oral CHM for migraine and explore the correlation between its effect size and treatment duration.

**Methods:**

We searched nine digital databases (PubMed, EMBASE, CINAHL, Cochrane Central Register of Controlled Trials, AMED, BioMedical Literature, CNKI, CQVIP, and Wanfang Data) from their inceptions to May 2021, with the language being restricted to Chinese and English. Randomized, placebo-controlled trials using oral CHM to treat adult migraine were included. Data screening and extraction were conducted by two independent reviewers. The methodological quality of randomized controlled trials (RCTs) was assessed using the Cochrane Risk of Bias tool. Meta-analyses were conducted to estimate the effect size using a random effect model, and a robust variance estimation (RVE) model was constructed to explore the correlation between treatment effects and treatment duration. The certainty of the evidence was assessed with the Grading of Recommendations Assessment, Development, and Evaluation. Publication bias was tested using a funnel plot and Egger's test.

**Results:**

A total of 18 RCTs involving 3,015 participants were included. Results of the meta-analyses showed that, at the end of the treatment phase, CHM was more efficacious than placebo in reducing migraine frequency, migraine days, and pain severity, and increasing response rate. Additionally, CHM showed superior effects to placebo in lowering migraine frequency and pain severity at the end of the 4-week follow-up. The RVE model suggested that the benefits of CHM for migraine frequency and pain intensity increased as treatment duration extended. The number of adverse events reported by the CHM and placebo groups was comparable. The certainty of the evidence was graded as “moderate.” No publication bias was detected.

**Conclusion:**

Oral CHM appeared to be more efficacious than placebo for reducing migraine frequency and pain severity. Greater treatment effects were associated with longer treatment duration. The oral CHM was well tolerated.

**Systematic Review Registration:**

https://www.crd.york.ac.uk/prospero/#recordDetails, identifier: CRD42021270719.

## Introduction

Migraine is a primary headache disorder that is characterized by recurrent, unilateral, and throbbing headaches and is associated with photophobia, phonophobia, nausea, and vomiting (1). Migraine was reported with a global age-standardized prevalence of 14.4% and induced 45.1 million years lived with disability (YLDs) in 2016 (2, 3). It was ranked as the first disabling disease for people aged under 50 according to the Global Disease Burden study 2016 (4). In China, migraine affected 150 million population and caused 5.5 million YLDs in 2017, as reported in a national-wide epidemiological study (5).

Conventional pharmacological therapies for migraine prevention include calcium channel blockers, beta-blockers, tricyclic antidepressants, selective serotonin reuptake inhibitors, calcitonin gene-related peptide (CGRP), etc. (6, 7). Flunarizine, a first-line prophylactic medication for migraine, is a selective calcium entry blocker with calmodulin binding properties and histamine H1 blocking activity (6–8). However, flunarizine is reported to be associated with side effects such as dizziness, weight gain, mood swings, etc. (9, 10), with an unsatisfying response rate estimated at 46.15% when it was used as a monotherapy (11). In China, over 60% of migraine patients seek Chinese medicine therapies, including Chinese herbal medicine (CHM), for migraine management (12). Research evidence demonstrated that oral CHM was comparable with or superior to conventional pharmacotherapies including flunarizine (13–15), and produced significant add-on effects when it was used in combination with conventional pharmacotherapies for migraine (13, 16). Two systematic reviews concluded that oral CHM was more efficacious than placebo as prophylactic management for migraine (15, 17). However, the sample size included in these reviews was limited, and the prolonged effects of CHM beyond the treatment phase were not evaluated.

Currently, the required treatment duration of migraine prophylactic medications remains controversial (18), although it was stated in clinical guidelines as “at least 6 weeks” (7) or “at least 3 months” (6). According to the Chinese Pharmacopeia, a few CHM products such as *du liang* pill, *tian shu* capsule, and *tou tong ning* capsule, are specifically used for migraine management, however, without a recommended treatment duration (19). Therefore, we conducted a comprehensive systematic review on randomized controlled trials (RCTs) comparing CHM with placebo to provide evidence on the efficacy of CHM for migraine prevention and used a robust variance estimation (RVE) model (20) to explore the correlation between treatment effects and treatment duration.

## Methods

This systematic review was conducted and reported in accordance with the requirements of the PRISMA 2020 (21) and the Cochrane Handbook (22).

### Data Source and Search Strategy

We searched nine electronic databases from their inceptions to May 2021, these are PubMed, Excerpta Medica Database (EMBASE), Cumulative Index of Nursing and Allied Health Literature (CINAHL), Cochrane Central Register of Controlled Trials (including the Cochrane Library), the Allied and Complementary Medicine Database (AMED), BioMedical Literature, China National Knowledge Infrastructure (CNKI), Chongqing VIP (CQVIP) and Wanfang database. The language was restricted to Chinese and English. The search terms consisted of four groups: participants' condition (adult migraine), intervention (CHM, Chinese patent medicine, formula^*^, and related terms), control (placebo), and study design (RCT) (See Supplementary File 1).

### Eligibility Criteria

RCTs meeting the following criteria were included: (1) adult participants (aged 18 years or above) being diagnosed with migraine; (2) oral CHM was used as the sole intervention; (3) only placebo was utilized in the control group; (4) routine cares and acute medications were allowed but should be identical in both groups; and (5) reporting any of the following outcomes: migraine attack frequency per month (4 weeks), number of migraine days per month (4 weeks), response rate (defined as the proportion of people who achieved a 50% reduction in migraine frequency), headache pain severity [measured by visual analog scale (VAS) or numeric rating scale (NRS)], the average duration of migraine attacks (hours), frequency of taking acute medication, days using an acute medication, and health-related quality of life. Migraine frequency per month (4 weeks) is selected as the primary outcome in this review, as recommended by the International Headache Society (IHS) Clinical Trials Committee in 2012 (23).

Studies were excluded if they were in any of these scenarios: (1) evaluation period being less than 4 weeks; (2) oral CHM and placebo were used in combination with other types of Chinese medicine therapies, or other migraine prophylactic treatments; and (3) duplicate publications reporting results from the same study.

### Study Screening and Data Extraction

Two reviewers (SL and CZ) performed study screening in two steps: (1) a preliminary screening on titles and abstracts and (2) further eligibility screening against selection criteria based on full text. Data extraction was conducted by one reviewer (SL) and checked by the second reviewer (CZ). Information on study characteristics, disease duration, details of intervention (CHM formula names, ingredients, and manufacturers), treatment duration, follow-up duration, outcome measures, and adverse events (AEs) were extracted from the eligible RCTs. Where there were missing, conflicting, or unclear data, the corresponding author of the study was contacted via email for further clarification.

### Risk of Bias Assessment

The methodological quality of included RCTs was assessed using the Cochrane Risk of Bias tool (22) by two independent reviewers (SL and CZ). Judgment was made in domains of random sequence generation, allocation concealment, blinding of participants and personnel, blinding of outcome assessment, incomplete outcome data, selective reporting, and other biases such as potential bias caused by funding source or conflict of interest. Studies were labeled as “high,” “unclear” or “low” risk of bias for each domain. The discrepancy between these two reviewers was resolved after consulting a third reviewer (XG).

### Synthesis of Results

Data analysis was performed using the R 4.0.5, Review Manager 5.3 and Stata 12.0 software. Mean difference (MD) with the estimated 95% confidence interval (CI) was presented for continuous data and relative risk (RR) with 95%CI for binary data. Heterogeneity among the RCTs was assessed by the inconsistency index statistic (*I*^2^). The random effects model was applied for meta-analyses.

Pooled meta-analyses were conducted for clinical outcome data reported at the end of treatment (EoT) and at the end of the follow-up (EoFU) phases. Subgroup analyses were conducted using the RVE model in robumeta package based on all the repeated measured continuous outcomes at mid-treatment timepoints, to explore the correlation between treatment effects and treatment duration.

Publication bias was evaluated using a funnel plot and Eggers' test based on the meta-analyses which included more than 10 RCTs (22).

### Certainty of Evidence Assessment

The certainty of evidence of the primary outcome measures was evaluated as high, moderate, low, or very low according to GRADE (Grading of Recommendations Assessment, Development, and Evaluation Working Group) (24), taking into consideration the risk of bias, inconsistency of results, indirectness of evidence, imprecision, and publication bias.

## Results

### Characteristics of the Included RCTs

**Figure 1:**
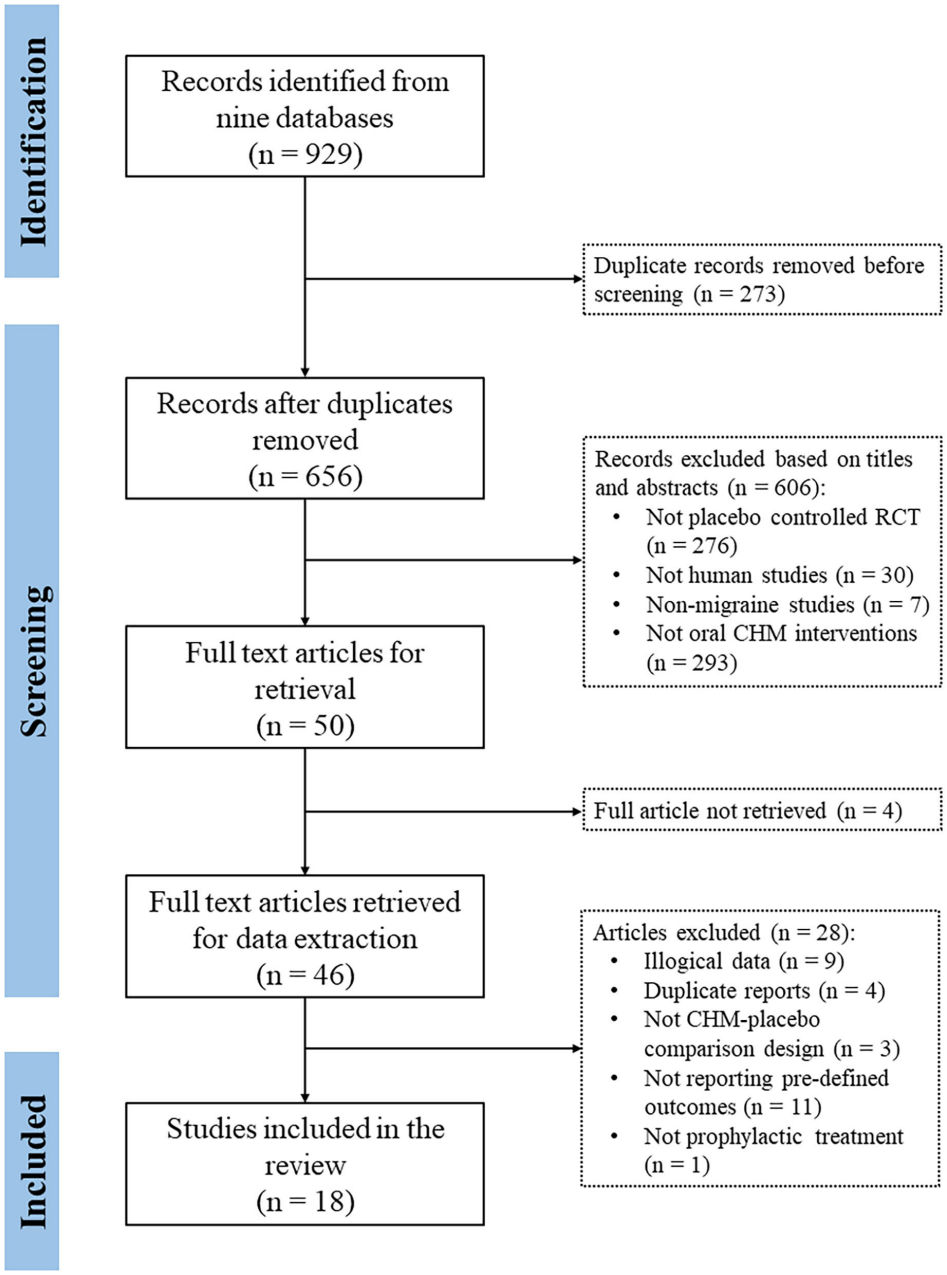
Flowchart of the research.

A total of 18 RCTs involving 3,015 participants (1,844 in the CHM group) were included in the systematic review (Figure 1). The characteristics of the included RCTs are presented in Table 1. Based on the available gender information, the number of female participants was over 2 times that of male participants (1,553 *vs*. 758). All participants were aged between 18 and 70 years old.

**Table 1:** Characteristics of included studies.

**Reference**	**No. of** **participants** **randomized** **(IG: CG)**	**Duration of** **migraine in** **IG (years)** **(Mean + SD)**	**Duration of** **migraine in** **CG (years)** **(Mean + SD)**	**Age in IG** **(years)** **Mean ±SD**	**Age in** **CG (years)** **Mean ±SD**	**Gender** **(F/M)**	**Treatment** **duration,** **follow-up** **duration** **(days)**	**Formula** **names** **(form)**	**Ingredients of** **formulae**	**Manufacturer of** **the medicine**	**Placebo**	**Acute** **medication** **in both** **groups**	**Routine** **cares in** **both** **groups**
Cao et al. (25)	109:110	5.3 ± 3.1	5.3 ± 3.1	38.57 ± 11.93	38.60 ± 11.56	168/51	84, 0	*Zheng tian* (pill)	*chuan xiong, qiang huo, fang feng, bai zhi, gou teng, tao ren, hong hua, dang gui, ji xue teng, di huang, du huo, fu zi, ma huang, xi xin, bai shao*	Huarun Sanjiu Pharmaceutical Co. Ltd. D	Placebo pill (ingredients: NA)	Ibuprofen and other analgesic drugs	Health education
Chen et al. (26)	120:60	16.21 ± 10.38	18.1 ± 9.02	41.98 ± 12.33	43.80 ± 11.03	NS	84, 28	*Xiong xie* (capsule)	*quan xie, wu gong*	Shanghai Baolong Pharmaceutical Co., LTD	Placebo capsule (1/20 *xiong xie* capsule and other unspecified ingredients)	Ibuprofen	NS
Fu et al. (27)	99:51	7.19 ± 7.34	6.84 ± 6.06	35.77 ± 11.60	34.58 ± 9.85	NS	84, 28	*Chuan xiong ding tong* (decoction)	*chuan xiong, niu xi, bi xie, ju hua, gou teng, bai ji li, yi yi ren, bai dou kou, ban xia*	Hua Run San-Jiu Pharmaceutical Co. LTD	Placebo decoction (dextrin, lactose, caramel pigment, and bitters)	NS	Health education lifestyle, emotion control and diet.
Ge (28)	65:21	NS	NS	NS	NS	NS	84, 0	*Chuan xiong* oil (soft capsule)	*chuan xiong*	NS	Placebo capsule (ingredients: NA)	NS	NS
Hu (29)	24:24	9.8 ± 7.3	8.2 ± 4	50 ± 9.8	46.2 ± 14.9	30/18	60, 0	*Zheng tian* (pill)	*chuan xiong, qiang huo, fang feng, bai zhi, gou teng, tao ren, hong hua, dang gui, ji xue teng, di huang, du huo, fu zi, ma huang, xi xin, bai shao*	Sanjiu Medical & Pharmaceutical Co., Ltd	Placebo pill (starch)	NS	NS
Li and Cui (30)	65:46	6.1	5.40	35.6	3.3	78/33	90, 0	*Jing tong ling* (capsule)	*tian ma, suan zao ren, zhen zhu mu, long chi*	Affiliated Hospital of Shandong Medical University	Placebo capsule (ingredients: NA)	NS	NS
Li (31)	107:107	7.5 ± 8.4	7.1 ± 6.9	41.7 ± 14.6	41.2 ± 12.1	NS	28, 28	*Chuan xiong qing nao* (granule)	*chuan xiong, dang gui, fang feng, bai zhi, mai dong, xi xin, qiang huo, du huo, cang zhu*	Jichuan Pharmaceutical Group Co., LTD	Placebo granule (ingredients: NA)	NS	NS
Luo et al. (32)	56:56	NS	NS	38.5 ± 8.6	37.6 ± 11	70/42	30, 0	*Yang xue qing nao* (granule)	*dang gui, chuan xiong, bai shao, shu di huang, gou teng, ji xue teng, xia ku cao, jue ming zi, zhen zhu mu, yan hu suo, xi xin*	Tianjin Tasly Pharmaceutical Co., LTD	Placebo granule (ingredients: NA)	NS	NS
Luo (33)	24:24	NS	NS	NS	NS	33/15	30, 0	*Xi feng zhi tong* (granule)	*dang gui, chuan xiong, bai shao, tian ma, bai zhi, xu chang qing, yan hu suo, xiang fu*	Guangxi Qiangshou Pharmaceutical Group Co. LTD	Placebo granule (ingredients: NA)	NS	NS
Mei (34)	20:20	NS	NS	NS	NS	25/15	56, 28	*Zheng tian* (pill)	*chuan xiong, qiang huo, fang feng, bai zhi, gou teng, tao ren, hong hua, dang gui, ji xue teng, di huang, du huo, fu zi, ma huang, xi xin, bai shao*	Sanjiu Medical & Pharmaceutical Co., Ltd	Placebo pill (ingredients: NA)	NS	NS
Ren et al. (35)	31:31	7.35 ± 5.13	8.42 ± 5.33	39.23 ± 8.93	34.41 ± 9.25	37/25	56, 0	*Tou tong ning* (capsule)	*tu fu ling, tian ma, he shou wu, dang gui, fang feng, quan xie*	Shandong Lukang Chenxin Pharmaceutical Co. LTD	Placebo capsule (ingredients: NA)	NS	NS
Wang et al. (36)	56:51	16.4 ± 2.2	6.7 ± 2.5	28.5 ± 5.7	27.8 ± 5.3	76/31	42, 0	*Du liang* (soft capsule)	*chuan xiong, bai zhi*	Chong qing Hua sen Pharmaceutical Co., LTD	Placebo capsule (starch)	NS	NS
Xu (37)	24:24	NS	NS	NS	NS	32/16	84, 0	*Pian tou tong* (granule)	*chuan xiong, niu xi, bi xie, ju hua, gou teng, bai ji li, yi yi ren, bai kou ren, fa ban xia*	Sanjiu Medical & Pharmaceutical Co., Ltd	Placebo granule (ingredients: NA)	NS	NS
Yang (38)	30:30	6.32 ± 2.80	6.25 ± 3.18	41.58 ± 12.5	40.23 ± 13.73	43/17	84, 0	*Shu feng zhi tong* (capsule)	*chuan xiong, bai zhi, wu zhu yu, bo he*	NS	Placebo capsule (ingredients: NA)	NS	NS
Yu et al. (39)	750:250	NS	NS	47.59 ± 11.86	47.82 ± 12.84	638/362	84, 0	*Tian shu* (capsule)	*chuan xiong, tian ma*	Kanion Pharmaceutical Company	Placebo capsule (starch 0.318 g, sunset yellow 0.003 g, Melanin 0.002 g, and *Tian shu* capsule pre-granulation intermediate 0.017 g)	NS	NS
Yu et al. (40)	200:200	9.55 ± 8.80	9.65 ± 8.31	38.48 ± 13.04	37.96 ± 13.33	290/110	28, 28	*Tou tong ning* (capsule)	*tu fu ling, tian ma, he shou wu, dang gui, fang feng, quan xie*	Buchang Pharmaceutical Company	Placebo capsule (ingredients: NA)	NS	Health education
Zhai (41)	42:14	NS	NS	NS	NS	33/23	28, 28	*Xi feng zhi tong* (granule)	*dang gui, chuan xiong, bai shao, tian ma, bai zhi, xu chang qing, yan hu suo, xiang fu*	Guangxi Qiangshou Pharmaceutical Group Co. LTD	Placebo granule (ingredients: NA)	Ibuprofen	Health education lifestyle, emotion control and diet.
Zhou et al. (42)	32:32	NS	NS	38.60 ± 10.40	36.9 ± 8.7	NS	30, 0	*Kai yu ning nao* (capsule)	NS	Pharmacy Department, Yantai Hospital of Traditional Chinese Medicine, Shandong Province	Placebo capsule (ingredients: NA)	NS	NS

*CG, control group; F, female; IG, intervention group; M, male; NA, not available; No., Number; NS, not specified; SD, standard deviation*.

Six RCTs were multi-center studies (25, 27, 30, 32, 39, 40) and the remaining 12 RCTs were completed in a single center (26, 28, 29, 31, 33–38, 41, 42). All RCTs compared oral CHM to placebo, with five studies providing the details of how the placebo was manufactured (26, 27, 29, 36, 40). In regards to co-interventions, six RCTs allowed participants to take acute pain medications (25–28, 32, 41), and four studies applied health education to both groups (25, 27, 39, 41). The treatment duration ranged from 4 weeks (31, 39, 41) or 30 days (32, 33, 42) to 12 weeks (25–28, 30, 37, 38, 40). Mid-treatment outcomes were reported in six studies with a per evaluation interval of 4 weeks (25, 27, 28, 30, 35, 40). Six RCTs reported outcomes at the end of the 4-week follow-up phase (26, 27, 31, 34, 39, 41). Fourteen RCTs reported data on the primary outcome measure (migraine frequency) at the EoT (25–29, 31, 34, 35, 37, 39–42) and six reported this at the EoFU (26, 27, 31, 39–41). The number of RCTs that reported secondary outcomes such as migraine days, pain VAS/NRS, migraine duration, frequency of acute medication, and the response rate was <10. In addition, 17 studies reported information on AEs, with eight RCTs mentioning that there were no AEs occurred during the study (29, 31, 33, 34, 37, 38, 41, 42), and nine RCTs reported AE details (25–28, 30, 32, 36, 39, 40).

Fourteen formulae were identified from the included RCTs, three of them were used in multiple studies: *zheng tian* pill (*n* = 3), *tou tong ning* capsule (*n* = 2), and *xi feng zhi tong* granule (*n* = 2). There were 37 herbs being prescribed in the 18 RCTs, among which- *chuan xiong* was the most frequently used herb (*n* = 13), followed by *dang gui* (*n* = 9), *bai zhi* (*n* = 8), *gou teng* (*n* = 6), *tian ma* (*n* = 5), and *fang feng* (*n* = 5). The most common herb pair was *chuan xiong* and *bai zhi* (*n* = 8), followed by *chuan xiong* paired with *dang gui* (*n* = 7) (Table 2).

**Table 2:** Most frequently used herbs in the included studies.

**Herb name** **in *Pinyin***	**Number. of** **studies**	**Scientific names**
*Chuan xiong*	13	Ligusticum chuangxiong Hort.
*Dang gui*	9	Angelica sinensis (Oliv.) Diels
*Bai zhi*	8	1. Angelica dahurica (Fisch. ex Hoffm.) Benth. et Hook. f. 2. Angelica dahurica (Fisch. ex Hoffm.) Benth. et Hook. f. var. formosana (Boiss) Shan et Yuan
*Gou teng*	6	Uncaria rhynchophylla (Miq.)Miq. ex Havil.
*Tian ma*	5	Gastrodia elata Bl.
*Fang feng*	5	Saposhnikovia divaricata (Turcz.) Schischk.

### Risk of Bias Assessment

**Figure 2:**
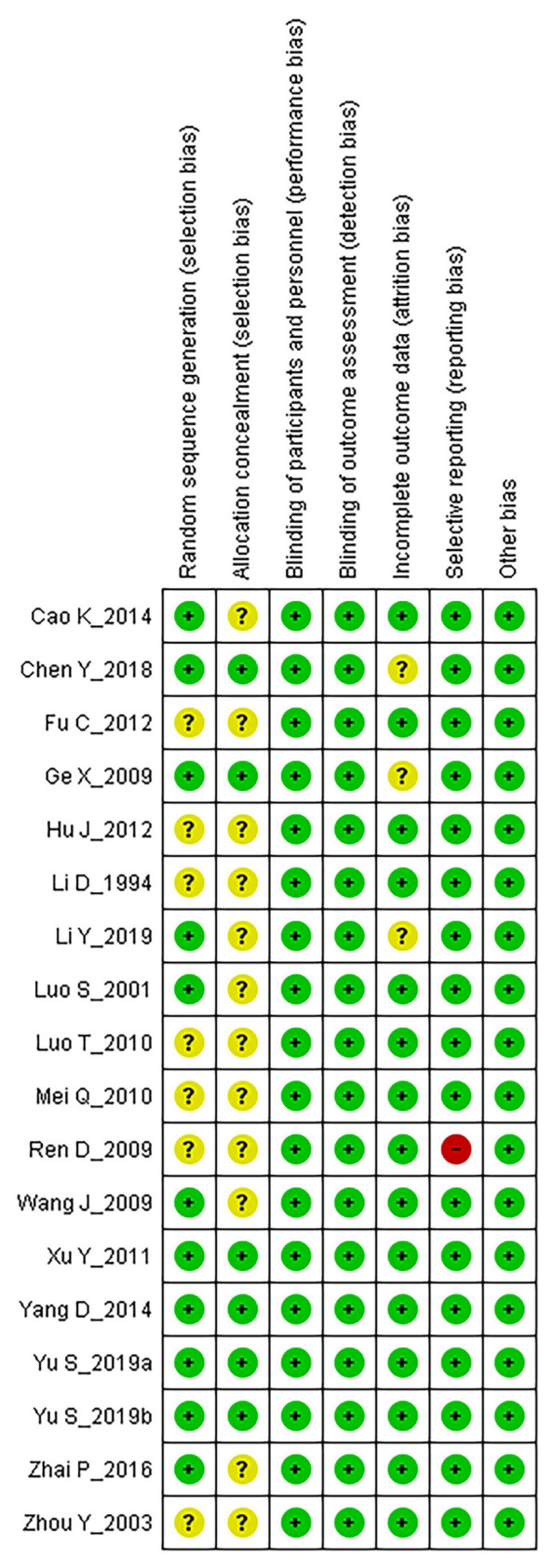
Risk of bias assessment.

The methodological quality of included RCTs was assessed using the Cochrane Collaboration risk of bias tool and presented in Figure 2. Regarding the random sequence generation, 11 RCTs were assessed as “low risk” for performing adequate randomization (25, 26, 28, 31, 32, 36–41), the remaining seven RCTs were “unclear” due to lack of information (27, 29, 30, 33–35, 42). Six RCTs depicted the allocation concealment method and were assessed as “low risk” for this domain (26, 28, 37–40), the remaining 12 studies were evaluated as “unclear” due to the absence of relevant information. All studies were assessed as “low risk” for blinding of participants, personnel, and outcome assessors since all these RCTs applied a proper placebo-controlled design. Regarding incomplete outcome data, three RCTs were categorized as “unclear” since they did not report reasons for dropouts nor applied any intention-to-treat analyses (26, 28, 31), the remaining RCTs were classified as “low risk.” One study did not report information on AEs, which was stated in the Methods section, and therefore it was assessed as “high risk” for selective reporting (35), the remaining 17 RCTs were assessed as “low risk” in this aspect. All RCTs were assessed as “low risk” for other biases since we did not detect any other biases such as conflict of interest and baseline imbalance.

### Primary Outcome Measures

#### Migraine Frequency

**Figure 3:**
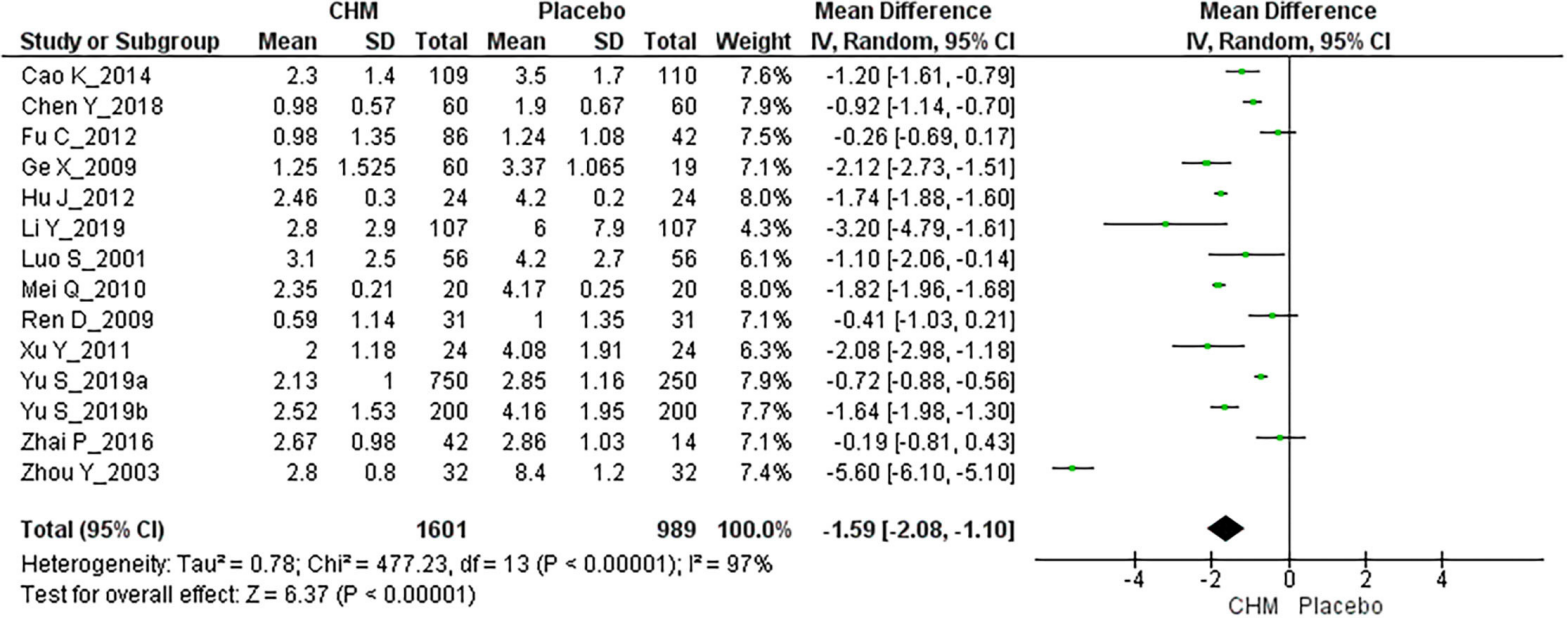
Meta-analysis for migraine frequency at the end of treatment.

Fourteen RCTs involving 2,590 participants reported migraine frequency at the EoT (25–29, 31, 34, 35, 37, 39–42). The overall meta-analysis showed a statistically significant benefit for CHM compared with placebo [MD: −1.59, 95% CI: (−2.08, −1.10), *I*^2^ = 97%] (Figure 3, Table 3).

**Table 3:** Treatment effects of all outcome measures.

**Outcome**	**Number of** **studies (*n* =)**	**Number of** **participants** **(IG/CG)**	**Estimated effects (RR or MD with 95% CI), *P-*value**	***I*^2^ (%)**
Frequency at the EoT	14	1,601/989	MD −1.59 (−2.08, −1.10), < 0.00001	97
Frequency at the EoFU	6	1,245/673	MD: −1.15 (−1.73, −0.56), 0.0001	94
Migraine days at the EoT	6	540/437	MD −1.93 (−2.75, −1.10), < 0.00001	86
Migraine days at the EoFU	2	286/242	MD: −186 (−4.19, 0.47), 0.12	95
Pain VAS/NRS at the EoT	9	1,294/725	MD: −1.19 (−1.59, −0.78), < 0.00001	86
Pain VAS/NRS at the EoFU	3	1,057/557	MD: −1.82 (−2.44, −1.20), < 0.00001	92
Attack duration at the EoT	6	488/489	MD: −4.05 (−8.12, 0.03), 0.05	98
Attack duration at the EoFU	2	242/214	MD: −1.18 (−3.07, 0.70), 0.22	87
Responder rate at the EoT	9	1,240/627	RR: 3.59 (2.01, 6.43), < 0.00001	90
Responder rate at the EoFU	3	1,036/492	RR: 2.26 (1.00, 5.11), 0.05	97
Frequency of taking analgesics at the EoT	3	878/306	MD: −0.29 (−0.67, 0.08), 0.12	85
Frequency of taking analgesics at the EoFU	2	836/292	MD: −0.43 (−0.89, 0.03), 0.06	92

*CG, control group; CI, confidence intervals; EoFU, end of follow-up; EoT, end of treatment; IG, intervention group; MD, mean difference; n, number; NRS, numeric rating scale; RR, risk ratio; VAS, visual analog scale*.

The advanced meta-analysis for migraine frequency using the RVE model indicated that CHM was not superior to placebo at end of the 4-week treatment [MD: −1.14, 95% CI (−2.52, 0.24), *I*^2^ = 97.85%]. While superior effect of CHM was detected after a 8-week treatment [MD: −0.86, 95% CI (−1.56, −0.16), *I*^2^ = 97.85%], and the difference increased when the treatment duration extended to 12 weeks [MD: −1.19, 95% CI: (−1.95, −0.42), *I*^2^ = 97.85%] (Table 4).

**Table 4:** Meta-analyses using robust variance estimation model with small-sample correction.

	**Migraine frequency** **MD (95% CI), *P-*value**	**Migraine days** **MD (95% CI), *P-*value**	**Pain VAS/NRS** **MD (95% CI), *P-*value**	**Migraine duration** **MD (95% CI), *P*-value**	**Frequency of taking** **analgesics** **MD (95% CI), *P*-value**
Week 4	−1.14 (−2.52, 0.24), 0.094	−0.74 (−2.58, 1.11), 0.328	−0.51 (−0.79, −0.23), 0.005^*^	−4.15 (−11.35, 3.05), 0.170	−0.18 (−1.05, 0.69), 0.412
Week 8	−0.86 (−1.56, −0.16), 0.023^*^	−1.15 (−2.53, 0.24), 0.084	−0.815 (−1.32, −0.30), 0.011^*^	−1.75 (−15.71, 12.21), 0.357	−0.21 (−3.32, 2.90), 0.554
Week 12	−1.19 (−1.95, −0.42), 0.010^*^	−1.73 (−3.88, 0.43), 0.084	−1.68 (−3.31, −0.06), 0.046^*^	−1.61 (−9.61, 6.39), 0.238	−0.29 (−3.06, 2.49), 0.416
I^2^	97.85%	91.56%	73.68%	97.95%	75.41%
Tau.sq	0.94	1.29	0.18	20.64	0.06
Omega.sq	0.06	0.27	0	0.95	0.03

*CI, confidence interval; MD, mean difference;*

* *significant at level of 0.05*.

**Figure 4:**
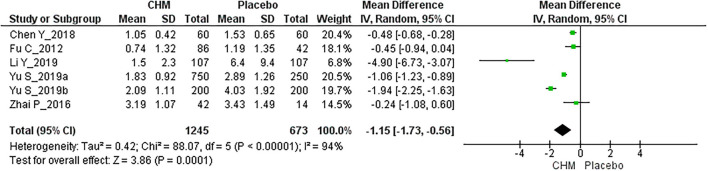
Meta-analysis for migraine frequency at the end of follow-up.

Six RCTs with 1,918 participants reported migraine frequency at the EoFU (26, 27, 31, 39–41), CHM achieved a greater reduction of migraine attacks than placebo [MD: −1.15, 95% CI (−1.73, −0.56), *I*^2^ = 94%] (Figure 4, Table 3).

### Secondary Outcomes

#### Migraine Days

Six RCTs involving 977 participants contributed to the meta-analysis of migraine days at the EoT (25, 27, 28, 30, 34, 39). Overall, CHM showed superior effects over placebo in reducing migraine days [MD: −1.93, 95% CI (−2.75, −1.10), *I*^2^ = 86%] (Table 3).

The analyses results using RVE model showed no significant between-group difference at mid-treatment timepoints regarding migraine days. Results at end of the 4^th^, 8^th^, and 12^th^ week was [MD: −0.74, 95% CI (−2.58, 1.11), *I*^2^ = 91.56%], [MD: −1.15, 95% CI: (−2.53, 0.24), *I*^2^ = 91.56%] and [MD: −1.73, 95% CI (−3.88, 0.43), *I*^2^ = 91.56%], respectively (Table 4).

Two RCTs with 528 participants reported migraine days at the EoFU. There was no difference between CHM and placebo [MD: −1.86, 95% CI (−4.19, 0.47), *I*^2^ = 95%] (27, 39) (Table 3).

#### Pain Intensity VAS/NRS

Pain VAS/NRS at the EoT was reported by nine studies involving 2,019 participants (26, 28, 29, 31, 34, 35, 39–41). The overall meta-analysis result indicated that CHM was more efficacious in alleviating pain when compared to placebo at the EoT [MD: −1.19, 95% CI (−1.59, −0.78), *I*^2^ = 86%] (Table 3).

The RVE model for pain VAS/NRS indicated that CHM achieved better pain relief than placebo at all three mid-treatment timepoints. The between-group difference of 4-week treatment was [MD: −0.51, 95% CI (−0.79, −0.23), *I*^2^ = 73.68%], it increased as treatment duration extended to 8 weeks [MD: −0.81, 95% CI: (−1.32, −0.30), *I*^2^ = 73.68%] and 12 weeks [MD: −1.68, 95% CI (−3.31, −0.06), *I*^2^ = 73.68%] (Table 4).

The superior pain relief effects of CHM at the EoFU were consistent with that during treatment. Three RCTs with 1,614 participants reported pain VAS/NRS at the end of the 4-week follow-up (31, 39, 40), and the meta-analysis result favored CHM [MD: −1.82, 95% CI (−2.44, −1.20), *I*^2^ = 92%] (Table 3).

#### Migraine Duration

Six RCTs involving 977 participants reported migraine duration at the EoT (25, 26, 32, 35, 39, 42), no statistical difference between CHM and placebo was found by the overall meta-analysis [MD: −4.05, 95% CI (−8.12, 0.03), *I*^2^ = 98%] (Table 3).

The results of analysis using the RVE model also showed no statistical between-group difference at three mid-treatment timepoints (Table 4).

Two RCTs reported average migraine duration at the EoFU (39, 41), and there was no difference between CHM and placebo [MD: −1.18, 95% CI (−3.07, 0.70), *I*^2^ = 87%] (Table 3).

#### Frequency of Taking Analgesic

Three RCTs reported the frequency of taking analgesics during the treatment phase (27, 40, 41). CHM did not show superiority in reducing the frequency of taking analgesics according to the meta-analysis [MD: −0.29, 95% CI (−0.67, 0.08), *I*^2^ = 85%] (Table 3).

The analyses results using RVE model indicated there was no statistical difference regardless of timing of evaluations. Results based on hybrid data after the 4-, 8- and 12-weeks treatment were [MD: −0.18, 95% CI (−1.05, 0.69), *I*^2^ = 75.41%], [MD: –0.21, 95% CI: (−3.32, 2.90), *I*^2^ = 75.41%], and [MD: −0.29, 95% CI (−3.06, 2.49), *I*^2^ = 75.41%], respectively (Table 4).

Similarly, according to the meta-analysis result based on two RCTs (27, 40), CHM was not different from placebo in terms of the frequency of taking analgesics at the EoFU [MD: −0.43, 95% CI (−0.89, 0.03), *I*^2^ = 92%] (Table 3).

#### Response Rate

There were nine RCTs involving 1,867 participants reporting response rates (27–29, 33, 37–41). The meta-analysis result indicated that CHM was more effective than placebo [3.59, 95% CI (2.01, 6.43), *I*^2^ = 90%] (Table 3).

The superiority of CHM was not sustained at the EoFU according to the meta-analysis on three RCTs of 1,528 participants [RR: 2.26, 95% CI (1.00, 5.11), *I*^2^ = 97%] (27, 39, 40) (Table 3).

### Heterogeneity

Significant heterogeneity was detected in the above-mentioned meta-analyses. We conducted sensitivity analyses on the outcome of migraine frequency by only including the studies of “low risk of bias” in randomization and allocation concealment, as well as subgroup analyses based on treatment duration and the formulae in the intervention group, respectively (Supplementary File 2). However, the heterogeneity cannot be reduced. The heterogeneity could be caused by various factors such as heterogeneous populations, adjustment of confounders, and different herbal compounds or dosages of interventions.

### Publication Bias

**Figure 5:**
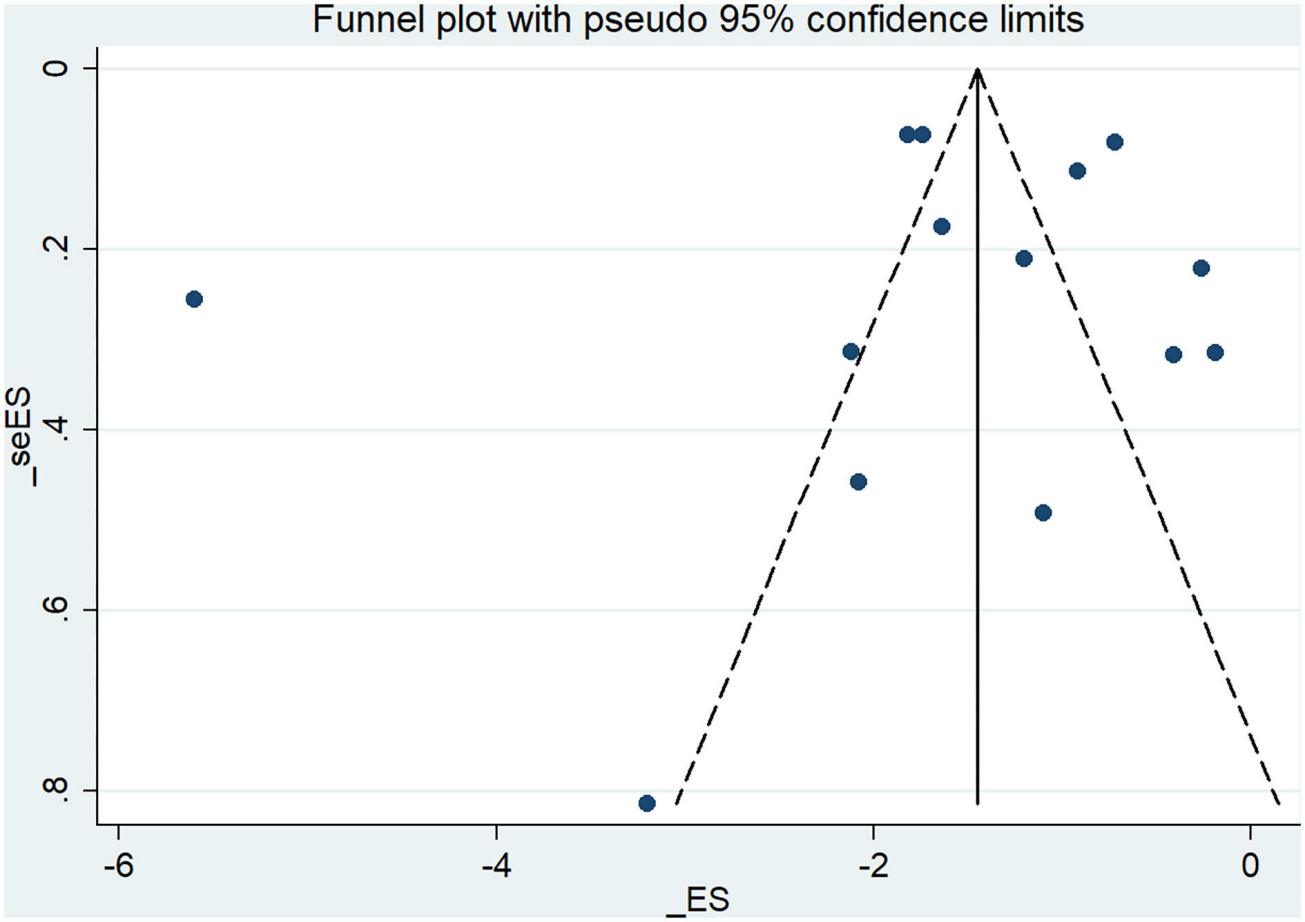
Funnel plot of RCTs reporting migraine frequency at the end of treatment.

The funnel plot based on the studies that reported migraine frequency at the EoT showed visual asymmetry (Figure 5), while Egger's test indicated that publication bias was not detected [*t* = –0.27, 95% CI (−7.07, 5.49)].

### Certainty of Evidence

Oral CHM was more efficacious than placebo for reducing migraine frequency at the EoT and EoFU, the certainty of this evidence was “moderate” based on GRADE assessment (Table 5).

**Table 5:** Summary of GRADE assessment.

**Outcomes**	**Number of** **participants**	**Number of** **studies**	**Estimated** **effects (MD** **with 95% CI)**	**Certainty of** **the evidence**
Migraine frequency at the end of treatment	2,590	14	−1.59 [−2.08, −1.10]	⊕⊕⊕◯ MODERATE ^a^
Migraine frequency at the end of follow–up	1,918	6	−1.15 [−1.73, −0.56]	⊕⊕⊕◯ MODERATE ^a^
**GRADE Working Group grades of evidence**				
**High certainty:** We are very confident that the true effect lies close to that of the estimate of the effect.				
**Moderate certainty:** We are moderately confident in the effect estimate: The true effect is likely to be close to the estimate of the effect, but there is a possibility that it is substantially different.				
**Low certainty:** Our confidence in the effect estimate is limited: The true effect may be substantially different from the estimate of the effect.				
**Very low certainty:** We have very little confidence in the effect estimate: The true effect is likely to be substantially different from the estimate of effect.				
**Explanation:** ^a^high heterogeneity may limit the certainty of the results.				

*CI, Confidence interval; MD, Mean difference*.

### Adverse Effects

There were 111 participants from the CHM group who reported 114 cases of AEs, and 80 participants from the placebo group reported 80 cases of AEs. No statistical difference was detected in the proportion of participants reporting AEs between the CHM and placebo groups [RR: 0.84, 95% CI (0.64, 1.11), *Z* = −1.20].

As presented in Table 6, the most common AEs in both CHM and placebo groups are gastrointestinal symptoms, followed by insomnia and somnolence. One participant withdrew from the placebo group due to severe nausea and chest discomfort (32). Another participant withdrew from the CHM group due to skin allergy, which required anti-allergy treatment (28). Other AEs were mild and resolved spontaneously without additional medical intervention.

**Table 6:** Summary of adverse events.

	**Number and severity** **reported by the CHM** **group**	**Number and severity** **reported by the** **placebo group**
Gastrointestinal symptoms	54 mild	45 mild, 1 moderate
Epigastric pain	10 mild	10 mild
Nausea	7 mild	4 mild, 1 moderate^*^
Diarrheal	7 mild	5 mild
Abdominal tympany	6 mild	6 mild
Constipation	6 mild	7 mild
Abdominal distention	6 mild	4 mild
Indigestion	5 mild	7 mild
Gast ectasia	4 mild	1 mild
Vomiting	2 mild	0
Stomachache	1 mild	1 mild
Dizziness	8 mild	3 mild
Insomnia	6 mild	5 mild
Somnolence	4 mild	5 mild
Abnormal liver function	3 mild	0
Dry mouth	3 mild	1 mild
Fatigue	2 mild	4 mild
Abnormal Urine leukocyte	2 mild	0
Acne	1 mild	0
Conjunctival congestion	1 mild	1 mild
Epistaxis	1 mild	1 mild
Hemoglobin level elevation	1 mild	0
Menometrorrhagia	1 mild	0
Platelet count elevation	1 mild	1 mild
Skin allergy	1 moderate	0
Chest discomfort	0	1 moderate^*^
AEs without detailed information	25 mild	14 mild
All adverse events reported in treatment and follow-up period	114 mild, 1 moderate	80 mild, 1 moderate

*CHM, Chinese herbal medicine;*

* *AEs reported by the same participant*.

## Discussion

### Summary of the Results

This study systematically reviewed 18 RCTs that compared CHM to placebo. As indicated by the meta-analyses, CHM was more efficacious than placebo in reducing migraine attack frequency and migraine days, relieving pain severity, and increasing response rate at the EoT. The superior efficacy of CHM at the EoFU was found in reducing migraine frequency, and reducing pain severity, but not in reducing migraine days or increasing response rate. In addition, there was no statistically significant difference between CHM and placebo in reducing the frequency of taking analgesics or shortening the average duration of migraine attacks at the EoT and at the EoFU based on limited data.

It should be pointed out that, the latest guideline published by IHS recommended using “migraine days” as the primary outcome for controlled trials in episodic migraine in adults (43). This systematic review was designed prior to the publication of the IHS 2020 guideline and selected “migraine frequency” as the primary outcome according to the recommendation of a guideline published by the IHS in 2012 (23). Considering all of the included studies were conducted before the publication of the IHS 2020 guideline, and most of them selected “migraine frequency” as the primary outcome measure, in order to respect the original design of the included RCTs and to maximize the data included in the efficacy analyses, we kept “migraine frequency” as the primary outcome in this review, and the “migraine days” as the secondary outcome measure.

The consumption of pain medication has raised great concerns because it may lead to medication overuse headache and transform episodic migraine into chronic migraine (44). It is worth noting that, meta-analyses results in this systematic review showed CHM was superior to placebo in reducing migraine attack frequency and pain severity but not on the frequency of taking analgesics. Similar results were found in previous studies (27, 45–48). As mentioned above in the migraine clinical trial guideline (23), a migraine attack can last for several days and consist of several episodes with various durations, each episode of a migraine attack can be temporarily relieved by acute medication. In addition, analgesic consumption is associated with headache history duration (49) and age (50). Therefore, the reduction in pain intensity, migraine frequency, and the times of acute medication consumption are not necessarily consistent. The impact factors of analgesic consumption and the effective method to reduce analgesic consumption deserve further exploration in future research. Furthermore, it has been recommended by recent research that, another outcome measure, namely “acute migraine-specific medication days” is usually consistent with migraine frequency, migraine days, and pain intensity (51–54). However, this specific outcome measure was not reported in the RCTs included in this review.

According to the RVE model analyses that taking all mid-treatment data into model construction, the statistical differences between CHM and placebo in migraine frequency was not seen at the 4-week timepoint, however, it was detected after 8 weeks and increased after 12-week treatment. Moreover, the significant superior effects of CHM for pain VAS also increased along with the extension of treatment duration. However, there was no statistical difference at any mid-treatment timepoint between CHM and placebo regarding migraine days, migraine duration, and frequency of taking analgesics. The inconsistency between pooled analysis results and RVE model results regarding migraine days could be caused by the fact that the results were calculated based on different datasets: six studies reported EoT data while only four of them reported mid-treatment data.

In summary, this study provides moderate-certainty evidence on the efficacy of CHM for migraine, especially in reducing migraine frequency and relieving pain severity. The results suggest that the treatment effects increased along with the extension of treatment duration, and the efficacy persists until 4 weeks after the cessation of CHM treatment. CHM is safe and does not induce a higher risk of AEs than placebo.

### Mechanism of Core Herbs for Migraine

*Chuan xiong* was the most popular used herb for migraine among the included RCTs, this is consistent with the findings of previous studies (14, 55–57). In addition, we found that *chuan xiong* and *bai zhi* and *chuan xiong* and *dang gui* were the two mostly used herb pairs for migraine management. Senkyunolide I, one of the active compounds of *chuan xiong*, was discovered to perform its anti-migraine activities by adjusting the levels of monoamine neurotransmitters and their turnover rates, as well as decreasing nitric oxide levels in the blood and brain (58). In addition, the volatile oil from *chuan xiong* presented an analgesic effect by inhibiting the *c*-fos gene expression and plasma CGRP in nitroglycerin-induced headache rats (59). The anti-migraine effects were enhanced when *chuan xiong* was paired with *bai zhi* and *dang gui* (60–63). These herb pairs have shown anti-migraine actions by reducing serum CGRP, serum nitric oxide, and brain dopamine, and increasing the levels of plasma endothelin, brain 5-hydroxytryptamine, and norepinephrine (60–63).

### Implication in Clinical Practice

In addition to previous systematic reviews comparing CHM to placebo (15, 17), our study provides moderate-certainty evidence to further confirm the efficacy and safety of CHM for migraine management and suggests the treatment duration of CHM should be extended to 8 or 12 weeks to increase clinical effects.

As a commonly used complementary therapy for migraine management in China (12), the benefits, safety, and economic cost induced by CHM remain in heated debates (12). Although the efficacy and effectiveness of CHM for migraine were supported by previous research (13–17), it remains inconclusive in terms of whether CHM should be used solely or in combination with conventional pharmacotherapies for migraine in real-world clinical practice, due to the low quality of the above evidence. In addition, it is widely recognized that preventive treatments should take patients' preferences, proven efficacy, and drug side effects into consideration in migraine management (18), which is consistent with the concept of evidence-based practice (64). More real-world evidence addressing patients' preferences and drug side effects is expected to support the evidence-based practice of CHM for migraine management.

### Strengths of This Study

In this review, we conducted parallel analyses both simply using final outcomes observed particularly at the EoT and analyses using the RVE model based on all longitudinal data (65). A discrepancy between the results of overall meta-analyses and the RVE model was noticed. The results generated by the latter method may be more reliable since the RVE model took the temporal non-independence between measurements into consideration (66, 67).

### Limitations of This Study

First, significant heterogeneity was detected and cannot be reduced by conducting subgroup analyses based on the risk of bias assessment, treatment duration, and CHM formulation. The heterogeneity could be caused by various factors such as heterogeneous populations and different herbal compounds or dosage of interventions. Second, the follow-up periods of the included RCTs were limited to 4 weeks, therefore, preventing us from drawing a conclusion on the long-term efficacy of CHM for migraine. Third, the diversity of herbal formulations used in the included clinical trials may limit the generalizability of the findings. In addition, in the IHS 2020 guideline, “migraine days” has been recommended as the primary outcome measure for clinical trials in episodic migraine. However, this outcome measure has not been reported as common as “migraine frequency” by the clinical trials included in this review.

## Conclusion

Oral CHM appeared to be generally efficacious and safe for migraine, particularly in reducing migraine frequency and pain severity. The efficacy increased as treatment duration extended from 4 weeks to 12 weeks and persisted until the EoFU. However, the evidence of CHM in reducing migraine days is insufficient, and the effects of reducing analgesic consumption need further evaluation. Oral CHM, in particular the herb *chuan xiong*, the herb pairs *chuan xiong*, and *bai zhi, chuan xiong*, and *dang gui*, should be considered as migraine prophylactic management in clinical practice. The findings of this systematic review warrant further validation by real-world research in the clinical practice setting.

## Data Availability Statement

The original contributions presented in the study are included in the article/Supplementary Material, further inquiries can be directed to the corresponding authors.

## Author Contributions

This paper was initially conceived and drafted by SL, CZ, AZ, JS, and XG. XL and CX contributed informative and critical comments in the manuscript revising. SL registered the study on PROSPERO and conducted the data search. SL and CZ screened potential studies, extracted data, drafted the manuscript, and evaluated the methodology quality of studies independently. SL and GC conducted data analyses. All authors approved of the final version of the manuscript. SL, CX, and XL undertook the final proofing of the manuscript and are fully responsible for the accuracy of the paper.

## Funding

This study was supported by the China-Australia International Research Center for Chinese Medicine. It was funded by Guangzhou University of Chinese Medicine Double First -Class and High-level University Discipline Collaborative Innovation Team (No. 2021xk84) and the Studio of Prof. Huang Huang based in Guangdong Provincial Hospital of Chinese Medicine (Grant No. E43723).

## Conflict of Interest

The authors declare that the research was conducted in the absence of any commercial or financial relationships that could be construed as a potential conflict of interest.

## Publisher's Note

All claims expressed in this article are solely those of the authors and do not necessarily represent those of their affiliated organizations, or those of the publisher, the editors and the reviewers. Any product that may be evaluated in this article, or claim that may be made by its manufacturer, is not guaranteed or endorsed by the publisher.
