# Supplementary material for: Efficacy and Safety of Oral Chinese Herbal Medicine for Migraine: A Systematic Review and Meta-Analyses Using Robust Variance Estimation Model
Source: Front Neurol. 2022 Jul 6;13:889336. doi: 10.3389/fneur.2022.889336 (PMC9296769; doi:10.3389/fneur.2022.889336)
Supplement: Supplementary file 1 [file Table_1.DOCX]

**Supplementary file 1: Search strategy in English language dataset**

Migraine AND (traditional Chinese medicine OR Chinese traditional medicine OR Chinese herbal drugs OR Chinese drugs, plant OR medicine, traditional OR ethnopharmacology OR ethnomedicine OR ethnobotany OR medicine, kampo OR kanpo OR TCM OR T.C.M. OR medicine, ayurvedic OR phytotherapy OR herbology OR plants, medicinal OR plant preparations OR plant extracts OR plants, medicine OR materia medica OR single prescription OR herbs OR Chinese medicine herb OR herbal medicine OR Chinese patent medicine OR formula*,) AND placebo AND (RCT OR randomized controlled trial OR randomised controlled trial)
