# Supplementary material for: Efficacy and Safety of Oral Chinese Herbal Medicine for Migraine: A Systematic Review and Meta-Analyses Using Robust Variance Estimation Model
Source: Front Neurol. 2022 Jul 6;13:889336. doi: 10.3389/fneur.2022.889336 (PMC9296769; doi:10.3389/fneur.2022.889336)
Supplement: Supplementary file 2 [file Table_2.DOCX]

# Supplementary file 2: Sensitivity analysis on migraine frequency at the end of treatment

|  | **Number of studies (n=)** | **Number of participants (I/C)** | **Estimated effects (MD with 95%CI, P value)** | **I^2^ (%)** |
| --- | --- | --- | --- | --- |
| Low risk of randomization | 9 | 1,408/840 | -1.28 (-1.66, -0.91), <0.00001 | 86 |
| Low risk of allocation concealment | 5 | 1,094/553 | -1.39 (-1.87, -0.92), <0.00001 | 91 |
| Four weeks’ treatment at the EoT | 5 | 437/409 | -2.34 (-4.44, -0.24), 0.03 | 98 |
| Eight weeks’ treatment at the EoT | 3 | 75/75 | -1.51 (-1.88, -1.14), <0.00001 | 89 |
| 12 weeks’ treatment at the EoT | 6 | 1,089/505 | -1.10 (-1.48, -0.71), <0.0001 | 86 |
| *Zheng tian* pill | 3 | 153/154 | MD: -1.67 (-1.90, -1.45) | 74 |
| *Tou tong ning* capsule | 2 | 231/231 | MD: -1.05 (-2.26, 0.15) | 91 |
| *Xi feng zhi tong* granule | 1 | 42/14 | MD: -0.19 (-0.81, 0.43) | N/A |

Note: C: control group; CI: confidence intervals; EoT: end of treatment; I: intervention group; MD: mean difference; n: number.
